# Correction of Hypothyroidism Seems to Have No Effect on Body Fat

**DOI:** 10.1155/2013/576794

**Published:** 2013-09-17

**Authors:** Okan Bakiner, Emre Bozkirli, Emine Duygu Ersozlu Bozkirli, Kursat Ozsahin

**Affiliations:** ^1^Department of Endocrinology and Metabolism Diseases, School of Medicine, Başkent University, 01250 Adana, Turkey; ^2^Department of Internal Medicine, School of Medicine, Başkent University, 01250 Adana, Turkey; ^3^Department of Family Medicine, School of Medicine, Başkent University, 01250 Adana, Turkey

## Abstract

*Aim*. We aimed to observe the effects of L-thyroxine replacement therapy on body fat content determined with various anthropometric methods and a bioelectrical impedance analysis method in patients with hypothyroidism. *Methods*. Forty-two women with naive autoimmune hypothyroidism were included. Also, 40 healthy participants were enrolled as a control group. Weight, body mass index, waist circumference, and subscapulary, suprailiac, femur, biceps, and triceps skin fold thicknesses were measured. Body fat percentages were calculated and body fat measurements were performed. Euthyroidism was maintained with L-thyroxine. At the 6th and 18th month, of therapy, measurements were reperformed. *Results*. Mean TSH levels were 57.49 ± 36.46 mIU/L in hypothyroid group and 1.94 ± 1.12 mIU/L in control subjects at admission. In hypothyroid patients, calculated body fat percentages were greater than those of the control subjects during follow-up. Body fat percentage of each hypothyroid case decreased at 6- and 18-month controls, but the decrements were statistically insignificant. Although skin fold thicknesses measured from all sites were observed to decline, only those obtained from femur and biceps showed a significant decrease (*P* = 0.03 and *P* = 0.01, resp.). *Discussion*. Correction of hypothyroidism did not cause any improvement in body weight and body fat percentage. The decrease in skin fold thicknesses might probably result from the reduction in subcutaneous mucopolysaccharide deposits.

## 1. Introduction

Hypothyroidism is a complex disorder characterized by an increase in body weight [1]. About 15–30% of hypothyroid patients are reported to be overweight and sufficient replacement therapy generally induces significant weight loss. However, 7% of the patients were observed to preserve their original weight in spite of the successful treatment [2]. In hypothyroid patients, body fat content is shown to increase in parallel with body weight. This effect is attributed to the reduction of lipid metabolism in hypothyroidism [3, 4]. Nevertheless, the expected decrease in body fat content is not observed with replacement therapy in a few studies [5, 6]. In a recent study, it was reported that L-thyroxine therapy resulted in an increase in body mass index (BMI) independently from thyroid-stimulating hormone (TSH) levels [7]. In another study, the decrease in body weight of hypothyroid patients treated with L-thyroxine was found to be related with the excretion of excess body water associated with the improvement of myxedema state instead of the change in body adiposity [8]. All these data caused requery of the effects of L-thyroxine replacement therapy on body adiposity in hypothyroid patients [9]. Although there are many methods to determine body fat content, BMI and waist circumference are widely used due to their easiness and low cost [10–12]. Measurement of skin fold thickness (SFT) is another simple method used for this purpose [13, 14].

Bioelectrical impedance analysis system (BIA) is an easy and a cheap method which helps to identify different body compartments: body lipid percentage, fat-free body mass, and total body fluid [15].

In this study, we aimed to observe the effects of midterm thyroid replacement therapy on body fat content determined with various anthropometric methods and a bioelectrical impedance method. 

## 2. Materials and Methods

### 2.1. Selection of Participants

This study was performed in a university hospital's endocrinology and metabolism diseases outpatient clinic and it was designed as a prospective and controlled study. As body fat content is known to be different within the two genders, only females were asked to participate. Subjects with hypothyroidism due to autoimmune thyroiditis who did not administer L-thyroxine replacement therapy before, with TSH levels greater than 10 mIU/L and aged between 18 and 50 years, were enrolled to the study. For the chronic autoimmune thyroiditis diagnosis, the criteria used were elevation of at least one of the antithyroglobulin or antithyroid peroxidase autoantibodies and/or shown ultrasonographic changes due to thyroiditis. Exclusion criteria for the study were (1) postmenopausal women, (2) smoking, (3) patients using drugs which may affect thyroid functions like lithium, amiodarone, steroids, beta blockers, or interferon, (4) patients using drugs which may affect body water-lipid homeostasis like diuretics or oral contraceptives, (5) patients with chronic renal failure, hepatic failure, congestive heart diseases, malnutrition, or malignant diseases, (6) pregnant women, and (7) patients with other known endocrine disorders. A control group with similarly aged healthy women volunteers was also enrolled to the study. This study was approved by Başkent University Institutional Review Board and Ethics Committee (Project no. KA 13/11) and supported by Başkent University research fund.

Patients who did not come to control visits, did not reach target TSH levels (0.45–4.12 mIU/L) [16], became pregnant, or used drugs which might affect the results during follow-ups were excluded from the study. 

### 2.2. Methods

After 12 hours of fasting, weight and height of the patients and controls were measured with a standard steelyard at the first visit. Body mass index was calculated as kg/m^2^. Waist circumference was measured at the midpoint between the iliac crest and lower rib margin. Subscapular, suprailiac, femur, biceps, and triceps skin fold thicknesses (SFT) were measured by a caliper. Body fat percentage (BFP) was calculated with the data obtained via using the methods suggested by Deurenberg, Lean, and Durnins for female gender [17–19]:

 BFP: 1.2 × BMI + 0.23 × age (year) − 5.4 (by Deurenberg for BMI),
 BFP: 0.439 × waist circumference (cm) + 0.221 × age (year) − 9.4 (by Lean for waist circumference),
 BFP: 1.33 (triceps SFT +  subrascapular SFT) − 0.013 (triceps SFT + subscapular SFT) − 2.5 (by Durnins for SFT).

Body fat measurements were performed by using a foot-to-foot pressure electrode bioelectrical impedance analysis contact system (BIA) (Tanita TBF-105, Tanita Corp., Tokyo, Japan). Before testing, subjects were required to adhere to the BIA testing guidelines: (1) not to eat or drink (especially caffeinated products) within 4 hours of the test, (2) not to consume alcohol within 48 h of the test, (3) to avoid intense exercise within 12 hours of the test, (4) not to take diuretics within 7 days of the test, and (5) to empty bladder within 30 minutes of the test. The procedure was performed while the subject was standing erect barefoot on the device's footpads and wearing light clothes. All measurements were performed by the same researcher. 

Thyroid-stimulating hormone and free thyroxine (fT_4_) levels were obtained from the venous blood sample of the forearm brachial veins and were studied with electrochemiluminescence immunoassay by using Abbott-Artitect analyzer (Chicago, IL, USA). Anti-thyroid peroxidase levels were studied with electrochemiluminesans immunoassay by using Modular E170 analyzer (Roche Diagnostic, Mannheim, Germany). Then, each patient was given 1.6 mcg/kg/day L-thyroxine in order to reach the target TSH level; the dosage was increased when indicated. The subjects were advised not to change their dietary and exercise habits and all were called for regular control every six weeks. At the 6th and 18th months of follow-up, body fat percentages were recalculated and TSH measurements were reperformed by the same researcher using the methods mentioned above. Body fat percentages of the patients and the control group calculated by the mentioned formulas and BIA during the initial 6- and 18- month visits were compared. The intragroup changes for each group were also calculated. The relation between the measured changes in BFP and body weight was investigated.

### 2.3. Statistical Analyses

Statistical analysis was performed using *SPSS software* (Version 17.0, SPSS Inc., Chicago, IL, USA). All the numerical data are expressed as mean values ± SD or as proportions. An assessment of the normality was done initially. For each continuous variable, normality was checked by Kolmogorov Smirnov and Shapiro-Wilk tests. Comparisons between continuous variables were applied with student's *t*-test or one-way ANOVA for normally distributed data and Mann-Whitney *U* test or Kruscal Wallis test for the data not-normally distributed. Prepost measures data were analyzed by paired *t*-test, Wilcoxon tests or repeated measure analyses. Correlations between variables were tested by Pearson's correlation test and Spearman correlation test. Correlation coefficients were interpreted as either excellent relationship *r* ≥ 0.91; good 0.90 ≤ *r* ≥ 0.71; fair 0.70 ≤ *r* ≥ 0.51; weak 0.50 ≤ *r* ≥ 0.31; little or none *r* ≤ 0.3 (ref). The level for statistical significance was predetermined at *P* < 0.05 for all tests.

## 3. Results

Forty-two patients as study group and 40 subjects as a control group were enrolled to the study. Thirty-one patients as a study group and 34 subjects as control group completed the study because of the exclusion criteria mentioned in Section 2.

The characteristics of the patients and control subjects at inclusion, at the 6th month, and at the 18th month of the study are shown in Tables 1 and 2.

Mean age of the patients was 39.13 ± 17.7 years. Mean TSH level at inclusion was 57.49 ± 36.46 mIU/L (minimum: 10.12, maximum: 100), at the 6th month 1.26 ± 1.35 mIU/L, and at the 18th month, 1.38 ± 1.09 mIU/L. Final mean L-thyroxine dose was 1.54 ± 0.76 *μ*cg/kg/day. 

Mean age of the control subjects was 36.28 ± 21.3 years. There was no significant difference between the patient group and the control group when mean ages were compared (*P* = 0.63). Mean TSH level at inclusion was 1.94 ± 1,12 mIU/L (minimum: 0.52 and maximum: 4.1), at the 6th month 1.76 ± 1.01 mIU/L, and at the 18th month 1.49 ± 0.8 mIU/L. There was evident difference between initial mean TSH levels of the study group and control group (*P* = 0.001), while there was no statistically significant difference at the 6th and the 18th months (*P* = 0.28 and 0.31, resp.).

The comparisons of hypothyroid- and control-group characteristics during follow-up were given in Table 3.

The female gender is classified into the following groups regarding body fat measurements performed with Tanita: the ones with BFP(%) below 15 are regarded as thin, those between 15 and 22 as normal, those between 23 and 26 as overweight, those between 27 and 32 as obese, and the ones over 32 as morbid obese [16]. Considering this information, our hypothyroid-cases are grouped as obese throughout the follow-up,with BFP values 32.09 ± 2.81 at initiation, 31.11 ± 2.56 at the 6th month, and 31.02 ± 2.77 at the 18th month. 

In hypothyroid group, the mean body weight showed a slight decrease which did not exhibit statistical significance (*P* = 0.36 at the 6th month and *P* = 0.34 at the 18th month).

Using the above-mentioned four methods, the BFP of each case at inclusion was compared with the value at the 6th and the 18th months. In the hypothyroid group, a slight decrease was observed but it was not significant (Table 1). 

During follow-up, SFT measured from all sites were observed to decline but only those obtained from femur and biceps showed a statistically significant decrease (*P* = 0.03 and *P* = 0.01, resp.) (Table 4).

## 4. Discussion

Hypothyroidism is known to cause an increase in body weight and fat content. The decrease in thermogenesis and fat tissue metabolism and fluid retention are considered to be responsible for this situation [2]. In this study, in spite of the fact that the participants achieved euthyroidism with adequate L-thyroxine replacement, we did not observe a significant decrease in body weight and body fat percentages. Our findings are compatible with the animal studies and human studies reporting statistically insignificant change in BFP following the treatment of hypothyroidism [20–22]. 

The expected decrease in weight and body fat percentage was not observed in our study. This issue may resulted from a few reasons; one of them may be the increase in orexigenic response which can be triggered by increased thermogenesis. The persistence of hypothyroid milieu in the tissues in spite of normalized TSH levels may well be another explanation. 

Although there was no difference by means of body weight, waist circumference, and BFP, there was a significant decrease in SFT measured with a caliper. This finding might be attributed to the decrease in subcutaneous mucopolysaccharides accumulation resulting from hypothyroidism [23].

This study has a few limitations. Our inability to determine the fat percentages with the gold standard methods, computerized tomography or magnetic resonance imaging, was one of them [24]. But every patient being the control of his own and that all measurements being performed by the same researcher, we hope it may be appreciated. Another limitation was the small number of patients included. However, our study results support current medical literature, and bigger studies with larger study groups are required to clarify these findings.

Due to the classification regarding body fat measurements performed with Tanita, our subjects are grouped as obese, although they are considered to be overweight for BMI [25]. This finding may be attributed to the increase in body fat resulting from hypothyroidism. 

In conclusion, in spite of the correction of hypothyroidism, at the 6th and 18th months of follow-up, our cases did not exhibit significant changes in terms of body weight, waist circumference, and BFP determined via the methods mentioned above. It was of interest that a significant decrease in SFT occurred. This condition might be explained by the reduction of subcutaneous mucopolysaccharides which were deposited during the hypothyroid state.

## Figures and Tables

**Table 1:** The characteristics of patients during follow-up.

	Initial (*n* = 42)	At the 6th month (*n* = 36)	P*	At the 18th month (*n* = 31)	P**
Body weight (kg)	68.76 ± 16.78	67.83 ± 15.58	0.36	64.06 ± 20.86	0.34
Waist circumference (cm)	86.90 ± 13.2	86.06 ± 12.1	0.34	85.08 ± 14.53	0.17
BMI (kg/m^2^)	28.40 ± 7.0	27.90 ± 6.30	0.34	27.16 ± 7.66	0.34
BFP (%) with TANITA	32.09 ± 2.81	31.11 ± 2.56	0.25	31.02 ± 2.77	0.34
BFP according to waist circumference (%)	39.83 ± 1.89	39.46 ± 1.69	0.34	39.06 ± 1.84	0.34
BFP according to BMI (%)	40.22 ± 2.43	39.72 ± 2.19	0.33	39.10 ± 2.83	0.34
BFP according to SFT (%)	27.00 ± 5.83	26.22 ± 5.34	0.11	27.20 ± 5.24	0.11

Not significant (*P* > 0.05).

P*: initial versus 6th month, P**: initial versus 18th month.

**Table 2:** The characteristics of control subjects during follow-up.

	Initial (*n* = 40)	At the 6th month (*n* = 38)	P*	At the 18th month (*n* = 34)	P**
Body weight (kg)	60.24 ± 21.38	60.89 ± 23.02	0.24	60.59 ± 22.21	0.63
Waist circumference (cm)	82.2 ± 14.1	83.0 ± 13.7	0.49	82.16 ± 12.7	0.24
BMI (kg/m^2^)	26.42 ± 4.1	25.31 ± 5.30	0.58	26.02 ± 5.16	0.29
BFP (%) with TANITA	28.07 ± 3.21	29.01 ± 4.56	0.34	28.91 ± 2.28	0.69
BFP according to waist circumference (%)	34.13 ± 2.04	34.97 ± 2.01	0.48	34.02 ± 2.83	0.87
BFP according to BMI (%)	35.81 ± 2.34	36.24 ± 2.87	0.21	35.88 ± 1.93	0.74
BFP according to SFT (%)	24.37 ± 4.89	24.48 ± 5.21	0.19	24.22 ± 4.64	0.18

Not significant (*P* > 0.05).

P*: initial versus 6th month, P**: initial versus 18th month.

**Table 3:** The comparison of two group characteristics during follow-up.

	Initial *P*	At the 6th *P*	At the 18th month *P*
Body weight (kg)	0.001	0.02	0.019
Waist circumference (cm)	0.021	0.03	0.024
BMI (kg/m^2^)	0.012	0.014	0.012
BFP (%) with TANITA	0.023	0.031	0.027
BFP according to waist circumference (%)	0.04	0.037	0.041
BFP according to BMI (%)	0.037	0.042	0.038
BFP according to SFT (%)	0.047	0.043	0.047

Not significant (*P* > 0.05).

**Table 4:** Comparison of the initial regional skinfold thickness (SFT) with the end of the study in hypothyroid patients.

SFT region	First visit	Last visit	*P *
Forearm	23.43 ± 3.18	23.09 ± 2.58	0.17
Arm	31.0 ± 5.48	29.81 ± 5.00	0.24
Femur	53.12 ± 6.82	51.37 ± 6.70	0.03
Abdominal	99.03 ± 14.04	97.87 ± 12.51	0.07
Biceps	10.21 ± 3.96	9.41 ± 3.75	0.01
Triceps	19.47 ± 6.70	18.13 ± 6.40	0.31
Subscapular	22.26 ± 8.12	21.20 ± 8.13	0.22
Suprailiac	17.53 ± 5.84	16.60 ± 5.71	0.09

Not significant (*P* > 0.05).

## References

[1] Zulewski H, Müller B, Exer P, Miserez AR, Staub J-J (1997). Estimation of tissue hypothyroidism by a new clinical score: evaluation of patients with various grades of hypothyroidism and controls. *Journal of Clinical Endocrinology and Metabolism*.

[2] Krotkiewski M (2002). Thyroid hormones in the pathogenesis and treatment of obesity. *European Journal of Pharmacology*.

[3] Miyakawa M, Tsushima T, Murakami H, Isozaki O, Takano K (1999). Serum leptin levels and bioelectrical impedance assessment of body composition in patients with Graves’ disease and hypothyroidism. *Endocrine Journal*.

[4] Seppel T, Kosel A, Schlaghecke R (1997). Bioelectrical impedance assessment of body composition in thyroid disease. *European Journal of Endocrinology*.

[5] Langdahl BL, Loft AG, Eriksen EF, Mosekilde L, Charles P (1996). Bone mass, bone turnover and body composition in former hypothyroid patients receiving replacement therapy. *European Journal of Endocrinology*.

[6] Wolf M, Weigert A, Kreymann G (1996). Body composition and energy expenditure in thyroidectomized patients during short term hypothyroidism and thyrotropin-suppressive thyroxine therapy. *European Journal of Endocrinology*.

[7] Ruhla S, Arafat AM, Osterhoff M (2012). Levothyroxine medication is associated with adiposity independent of TSH. *Experimental and Clinical Endocrinology & Diabetes*.

[8] Karmisholt J, Andersen S, Laurberg P (2011). Weight loss after therapy of hypothyroidism is mainly caused by excretion of excess body water associated with myxoedema. *Journal of Clinical Endocrinology and Metabolism*.

[9] Kyle LH, Ball MF, Doolan PD (1966). Effect of thyroid hormone on body composition in myxedema and obesity. *New England Journal of Medicine*.

[10] World Health Organization Obesity (1997). Preventing and managing the global epidemic. *Report of A WHO Consulation on Obesity*.

[11] Calle EE, Thun MJ, Petrelli JM, Rodriguez C, Heath CW (1999). Body-mass index and mortality in a prospective cohort of U.S. adults. *New England Journal of Medicine*.

[12] Han TS, van Leer EM, Seidell JC, Lean ME (1995). Waist circumference action levels in the identification of cardiovascular risk factors: prevalence study in a random sample. *British Medical Journal*.

[13] Bray GA, Gray DS (1988). Treatment of obesity: an overview. *Diabetes/Metabolism Reviews*.

[14] Seidell JC, Cigolini M, Charzewska J, Ellsinger B-M, Deslypere JP, Cruz A (1992). Fat distribution in European men: a comparison of anthropometric measurements in relation to cardiovascular risk factors. *International Journal of Obesity*.

[15] Swartz AM, Evans MJ, King GA, Thompson DL (2002). Evaluation of a foot-to-foot bioelectrical impedance analyser in highly active, moderately active and less active young men. *British Journal of Nutrition*.

[16] Garber JR, Cobin RH, Gharib H (2012). American Association of Clinical Endocrinologists and American Thyroid Association Taskforce on Hypothyroidism in Adults. Clinical practice guidelines for hypothyroidism in adults: cosponsored by the American Association of Clinical Endocrinologists and the American Thyroid Association. *Thyroid*.

[17] Lean ME, Han TS, Deurenberg P (1996). Predicting body composition by densitometry from simple anthropometric measurements. *American Journal of Clinical Nutrition*.

[18] Durnin JV, Womersley J (1974). Body fat assessed from total body density and its estimation from skinfold thickness: measurements on 481 men and women aged from 16 to 72 years. *British Journal of Nutrition*.

[19] Deurenberg P, Weststrate JA, Seidell JC (1991). Body mass index as a measure of body fatness: age- and sex-specific prediction formulas. *British Journal of Nutrition*.

[20] Karakoc A, Ayvaz G, Taneri F (2004). The effects of hypothyroidism in rats on serum leptin concentrations and leptin mRNA levels in adipose tissue and relationship with body fat composition. *Endocrine Research*.

[21] Krassas GE, Pontikides N, Loustis K, Koliakos G, Constantinidis T, Kaltsas T (2006). Resistin levels are normal in hypothyroidism and remain unchanged after attainment of euthyroidism: relationship with insulin levels and anthropometric parameters. *Journal of Endocrinological Investigation*.

[22] Iglesias P, Alvarez Fidalgo P, Codoceo R, Díez JJ (2003). Serum concentrations of adipocytokines in patients with hyperthyroidism and hypothyroidism before and after control of thyroid function. *Clinical Endocrinology*.

[23] Smith TJ, Bahn RS, Gorman CA (1989). Connective tissue, glycosaminoglycans, and diseases of the thyroid. *Endocrine Reviews*.

[24] Rossner S, Bo WJ, Hiltbrandt E (1990). Adipose tissue determinations in cadavers—a comparison between cross-sectional planimetry and computed tomography. *International Journal of Obesity*.

[25] Lee RD, Nieman DC (1993). *Anthropometry: Nutritional Assessment*.

